# Primary human organoids models: Current progress and key milestones

**DOI:** 10.3389/fbioe.2023.1058970

**Published:** 2023-03-03

**Authors:** Giuseppe Calà, Beatrice Sina, Paolo De Coppi, Giovanni Giuseppe Giobbe, Mattia Francesco Maria Gerli

**Affiliations:** ^1^ Division of Surgery and Interventional Science, Department of Surgical Biotechnology, University College London, London, United Kingdom; ^2^ Stem Cell and Regenerative Medicine Section, Zayed Centre for Research into Rare Disease in Children, Great Ormond Street Institute of Child Health, University College London, London, United Kingdom; ^3^ Politecnico di Milano, Milano, Italy; ^4^ Specialist Neonatal and Paediatric Surgery, Great Ormond Street Hospital for Children NHS Foundation Trust, London, United Kingdom

**Keywords:** organoids, three dimensional model, regenerative medicine, primary tissue culture, disease modelling, primary organoids

## Abstract

During the past 10 years the world has experienced enormous progress in the organoids field. Human organoids have shown huge potential to study organ development, homeostasis and to model diseases *in vitro*. The organoid technology has been widely and increasingly applied to generate patient-specific *in vitro* 3D cultures, starting from both primary and reprogrammed stem/progenitor cells. This has consequently fostered the development of innovative disease models and new regenerative therapies. Human primary, or adult stem/progenitor cell-derived, organoids can be derived from both healthy and pathological primary tissue samples spanning from fetal to adult age. The resulting 3D culture can be maintained for several months and even years, while retaining and resembling its original tissue’s properties. As the potential of this technology expands, new approaches are emerging to further improve organoid applications in biology and medicine. This review discusses the main organs and tissues which, as of today, have been modelled *in vitro* using primary organoid culture systems. Moreover, we also discuss the advantages, limitations, and future perspectives of primary human organoids in the fields of developmental biology, disease modelling, drug testing and regenerative medicine.

## Introduction

**FIGURE 1:**
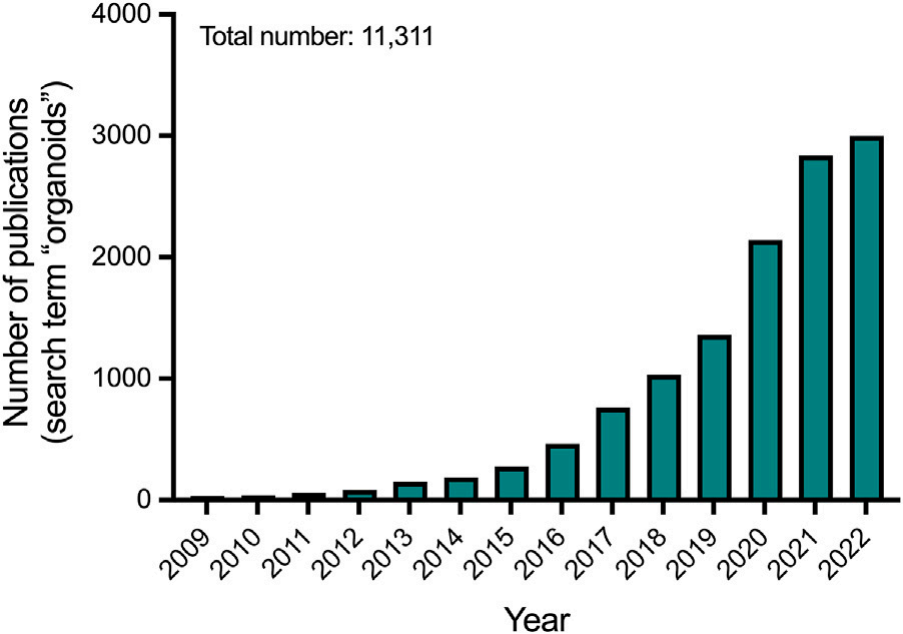
Number of publications per year on organoids according to Pubmed.

Organoids are three-dimensional structures that self-organize *in vitro*, recapitulating the microarchitecture and physiology of the tissue of origin. In 2009, Sato and colleagues’ led breakthrough work to develop a 3D culture system which enabled the growth of mouse intestinal epithelial organoids from single *Lgr5* ^+^ stem cells (Sato et al., 2009). These “mini guts” can be expanded and differentiated *in vitro,* to greatly resemble the intestinal tissue architecture and function. Since this seminal report, several protocols have been developed to establish human organoid lines, either from pluripotent or from tissue-resident stem cells (Clevers, 2016; Fatehullah et al., 2016) (Figure 1). Beside their translational potential for regenerative medicine applications, organoids are now widely used as an *in vitro* tool to investigate the development and function of most human tissue compartments. Organoids possess several advantages compared to traditional 2D culture systems. Overall, they display higher cellular heterogeneity, organization, and tissue-like structures, which make them more relevant *in vitro* model for functional analyses or personalised therapies (Li et al., 2019; Corrò et al., 2020). Consequently, organoids represent a unique experimental model to facilitate the investigation of the mechanisms underlying hereditary and acquired diseases, as well as developing patient-specific drug screening (Rossi et al., 2018; Tam et al., 2022). In this review, we outline the current progresses in the field of primary human stem/progenitor cells-derived epithelial organoids, with focus on their bioengineering applications, highlighting organoid-associated culture, analysis, and applications.

## Primary organoids derivation and culture

**FIGURE 2:**
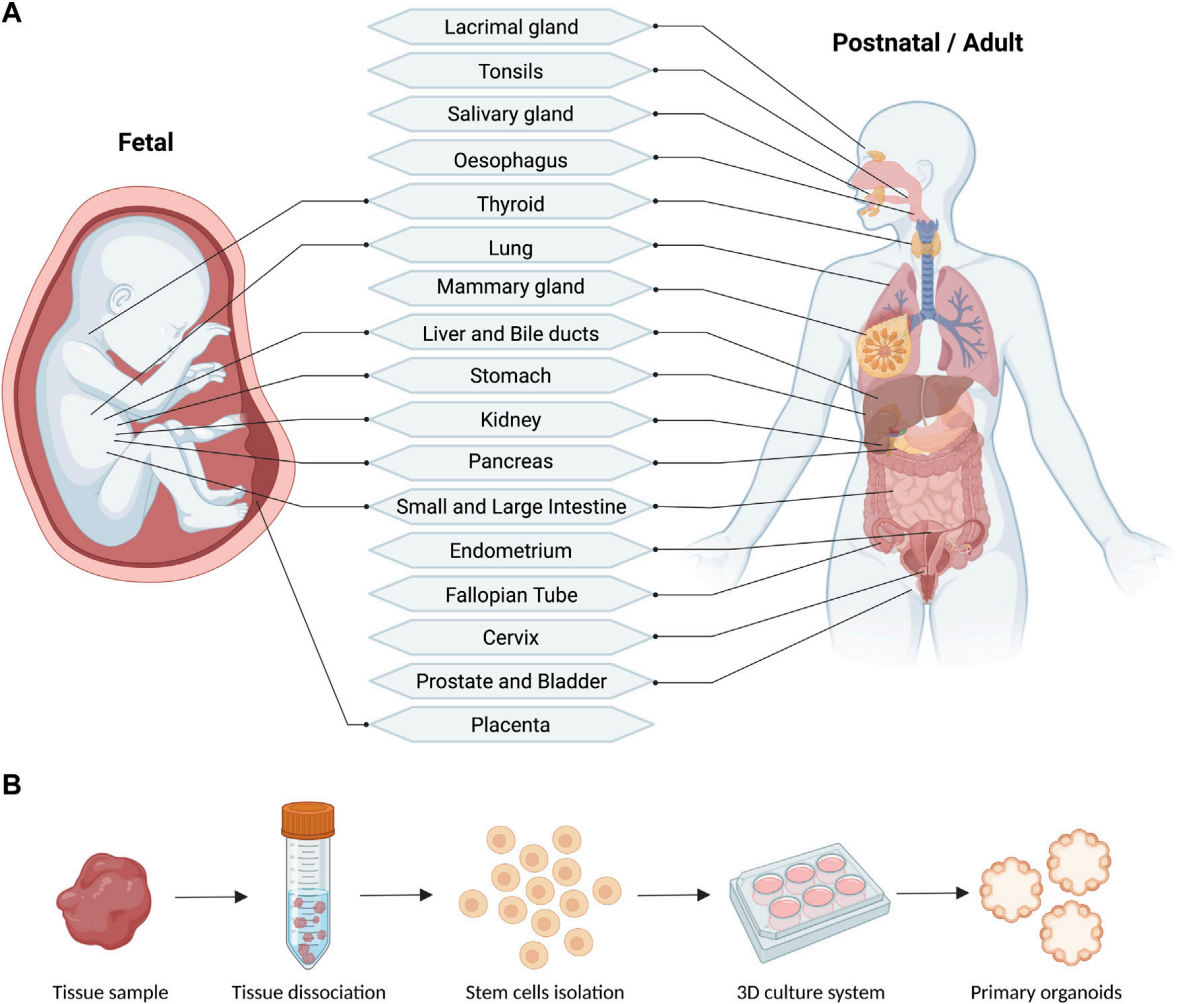
Schematic **(A)** and workflow **(B)** of the reported fetal and postnatal/adult tissues used for the derivation of primary human organoids (generated with BioRender).

To date, primary organoids have been derived to a large number of fetal and adult human tissues (Figure 2A). In general terms, three main components are required to establish a stable line of primary organoids: I) A human tissue sample (e.g., biopsies, surgical specimens, or fetal material); II) A cytocompatible and protein-rich embedding agents mimicking the tissue’s extracellular matrix (ECM); III) A growth media, rich in small molecules and growth factors able to maintain stemness and to induce tissue-specific processes. Following tissue disaggregation, cell isolation and 3D culture, single stem/progenitor cells start to divide and form organoids (Figure 2B). The organoids can be maintained in culture and passaged by mechanical or enzymatic dissociation to generate stable self-renewing lines. Most of the organoids derived from human primary samples mainly consist of different epithelial cells, arranged in a structure which morphologically and functionally mimics the primary tissue of origin. The culture condition for each tissue-specific stem/progenitor cell, aims at replicating the signals present in the niche from which the cells originate. A list of the culture conditions utilised to culture primary organoids of different human tissues is presented in Table 1. In this context, the extracellular matrix (e.g., ECM hydrogels, synthetic gels) is important to mimic, although not perfectly, the microenvironment of the tissues to enable efficient growth while providing trophic support. Wnt/b-catenin signalling has been described as the major driver of growth and plasticity of adult epithelial stem cells. Therefore, Wnt ligands such as Wnt-3a and R-Spondin one are key factors for growing epithelial organoids (Nusse and Clevers, 2017). This idea is also supported by the fact that many types of epithelial organoids are derived from LGR5+ stem cells that, upon activation of Wnt signalling, are able to divide clonally and subsequently differentiate to give rise to mature cell types within the organoids (Barker et al., 2010; Huch et al., 2013a; Huch et al., 2013b; Yin et al., 2013). Other important medium constituents for growing primary organoids are: serum- and xeno-free supplements, Wnt activators such as the GSK3-inhibitor CHIR, activators of tyrosine kinase receptor signalling such as EGF, and inhibitors of BMP/TGF-β signalling such as Noggin or A83-01. Specific growth factors and small molecules such as FGF family members (e.g., FGF7, FGF10), HGF and nicotinamide, are supplemented to the culture medium in a tissue-specific way. As one example, FGF7 and FGF10 were found to be important in promoting lung organoid morphogenesis and self-renewal (Nikolić et al., 2017). These components allow the maintenance of the stem cell niche, supporting proliferation and growth of the organoids in three dimensions, and their subsequent passaging. To ultimately induce the production of specific differentiated cells, some types of organoids may require withdrawal or addition of factors into their medium. Overall, chemically defined culture conditions are considered the most suitable and reproducible way to grow organoids and use them to develop GMP-compliant therapies. On the other hand, bioengineered cultures are paving the way to improve the reproducibility of organoid generation and to achieve real-world applications such as drug screening or clinical translation (Table 2). Lastly, the characterization of organoids is important to demonstrate their molecular similarity to native tissue. To this end, RNA profiling techniques (e.g., bulk, and single-cell RNA sequencing) are the most applied due to their broad, sensitive, and quantitative nature (Table 3).

**TABLE 1:** Summary of relevant human tissue-derived organoid models.

Organ	Age	Disease model	Medium supplements	Key findings	Ref
Lung	Fetal	-	EGF, FGF7, FGF10, Noggin, R-spondin, CHIR99021, SB431542	Derivation and differentiation of embryonic/fetal lung organoids that self-renew	Nikolić et al. (2017)
	Adult	SARS-CoV-2 infection	EGF, FGF7, FGF10, Noggin, R-spondin, CHIR99021, SB431542	SARS-CoV-2 infects AT2 cells in organoids	Youk et al. (2020)
	Adult	Cystic fibrosis, lung cancer, RSV infection, Primary cilia dyskinesia	FGF7, FGF10, Noggin, R-spondin, CHIR99021, A83-01, SB202190	Derivation, disease modelling, and gene editing of adult airway organoids	Sachs etal. (2019), Vaart et al. (2021)
	Adult	Influenza infection	FGF7, FGF10, Noggin, R-spondin, CHIR99021, A83-01, SB202190, Neuregulin-1	Differentiated airway organoids can predict infectivity of influenza viruses	Zhou et al. (2018)
	Adult	SARS-CoV-2 infection	EGF, Noggin, A83-01	Validation of a new progenitor cell type using distal lung organoids	Salahudeen et al. (2020)
	Adult	HSV and HPV infection, head and neck cancer	EGF, FGF10, FGF2, Noggin, R-spondin, A83-01, Prostaglandin E2, CHIR99021, Forskolin	Patient-derived mucosal organoids recapitulate HNSCC	Driehuis et al. (2019)
Tonsil	Adult	SARS-CoV-2 infection	FGF10, FGF2, HGF, Noggin, R-spondin, A83-01, Prostaglandin E2	Derivation of organoids from human tonsil tissue as a model for SARS-CoV-2 infection	Kim et al. (2022)
Oesophagus	Adult	Barrett’s oesophagus	EGF, FGF10, Wnt-3a, Noggin, R-spondin, Gastrin, A83-01, SB202190	Patient-derived oesophageal organoids for the disease modelling of Barrett’s oesophagus	Sato et al. (2011), Jiang et al. (2017)
	Adult	Eosinophilic esophagitis	EGF, Bovine pituitary extract, CaCl_2_	Oesophageal organoids can be used to model inflammatory conditions	Kasagi etal. (2018)
Stomach	Adult	*Helicobacter pylori* infection	EGF, FGF10, Wnt-3a, Noggin, R-spondin, Gastrin, A83-01	Derivation and disease modelling of adult gastric organoids	Bartfeld et al. (2015)
	Adult	*Salmonella enterica* and *Listeria* monocytogenes infection	EGF, FGF10, Wnt-3a, Noggin, R-spondin, Gastrin, A83-01, SB202190, CHIR99021	Gastric organoids with reversed apical-basal polarity for infectious disease modelling	Co et al. (2019)
	Fetal, Pediatric, Adult	SARS-CoV-2 infection	EGF, FGF10, Wnt-3a, Noggin, R-spondin, Gastrin, A83-01, CHIR99021	Reserved polarity and SARS-CoV-2 infection of fetal, paediatric, and adult gastric organoids	Giobbe etal. (2021), Jones et al. (2021)
Small and Large Intestine	Adult	Adenoma, colorectal cancer	EGF, Wnt-3a, Noggin, R-spondin, Gastrin, A83-01, SB202190	Generation of intestinal organoids from infected, inflammatory, or neoplastic tissues	Sato etal. (2011)
	Adult	-	EGF, Wnt-3a, Noggin, R-spondin, Gastrin, LY2157299, SB202190, Prostaglandin E2	Derivation of colonic epithelial organoids	Jung et al. (2011)
	Fetal	-	EGF, Wnt-3a, Noggin, R-spondin, A83-01, SB202190, Prostaglandin E2	Transplanted organoids derived from fetal intestinal epithelium can regenerate colon after injury	Fordham etal. (2013)
	Fetal	-	EGF, Wnt-3a, Noggin, R-spondin, A83-01, SB202190	Single cell profiling of fetal intestinal organoids	Elmentaite etal. (2020)
	Pediatric, Adult	Cystic fibrosis	EGF, Wnt-3a, Noggin, R-spondin, Gastrin, A83-01, SB202190	Intestinal organoids can be used to study and repair CFTR gene mutations	Dekkers etal. (2013), Schwank etal. (2013)
	Adult	Cryptosporidium infection	EGF, Wnt-3a, Noggin, R-spondin, Gastrin, A83-01, SB202190, Prostaglandin E2	Cryptosporidium infects and replicates in small intestinal and lung organoids	Heo et al. (2018)
	Adult	SARS-CoV-2 infection	EGF, Wnt-3a, Noggin, R-spondin, Gastrin, A83-01, SB202190	Infection of intestinal organoids suggests that the intestinal tract might be a transmission route of SARS-CoV-2	Zhou et al. (2020)
	Adult	*Salmonella enterica* and *Listeria* monocytogenes infection	EGF, Wnt-3a, Noggin, R-spondin, Gastrin, A83-01, SB202190, CHIR99021	Gastrointestinal organoids can model pathogen-epithelium interactions	Co et al. (2019)
	Fetal, Pediatric	-	EGF, Wnt-3a, Noggin, R-spondin, Gastrin, A83-01, SB202190, CHIR99021, Prostaglandin E2	Intestinal ECM-derived hydrogels as a 3D scaffold for growing fetal and adult intestinal organoids	Giobbe etal. (2019)
	Adult	-	EGF, Wnt-3a, Noggin, R-spondin, Gastrin, A83-01, SB202190, Prostaglandin E2	Bioprinted gastrointestinal organoids form centimetre-scale micro tissue	Brassard etal. (2020)
	Pediatric	Intestinal failure	EGF, Wnt-3a, Noggin, R-spondin, Gastrin, A83-01, SB202190, CHIR99021	Intestinal organoids from IF patients repopulate decellularized intestinal scaffold that can be transplanted	Meran etal. (2020)
Liver and Bile Ducts	Adult	α1-antitrypsin deficiency, Alagille syndrome	EGF, FGF10, Wnt-3a, Noggin, R-spondin, Gastrin, HGF, A83-01, Forskolin	Liver organoids can model both biliary duct and hepatocyte related disorders	Huch etal. (2015)
	Fetal, Pediatric, Adult	-	EGF, FGF7, FGF10, R-spondin, HGF, TGF-a, Gastrin, A83-01, CHIR99021	Derivation of organoids from single healthy hepatocytes	Hu et al. (2018)
Extrahepatic bile ducts	Adult	-	EGF, FGF10, HGF, Noggin, R-spondin	Derivation of organoids from gallbladder	Lugli etal. (2016)
	Adult	-	EGF, R-spondin, DKK-1, dexamethasone	Extrahepatic cholangiocytes organoids regenerate bile ducts	Sampaziotis et al. (2017)
Pancreas	Fetal	-	EGF, FGF10, Noggin, R-spondin, Gastrin	Derivation of organoids from fetal pancreatic progenitors	Bonfant etal. (2015)
	Fetal, Adult	-	EGF, FGF10, Noggin, R-spondin, Gastrin, A83-01	Derivation of adult pancreatic ductal organoids	Loomans etal. (2018)
Prostate and Bladder	Adult	-	EGF, FGF2, Noggin, R-spondin, A83-01, SB202190, dihydrotestosterone, Prostaglandin E2	Luminal epithelial cells can grow into prostate organoids	Karthaus etal. (2014)
	Adult	Bladder cancer	FGF2, FGF7, FGF10, A83-01	Derivation of bladder organoids and disease modelling of the urothelial cancer	Mullenders etal. (2019)
Thyroid	Adult	-	EGF, FGF2, Wnt-3a, Noggin, R-spondin, A83-01, Heparin	Adult thyroid organoids can form a functional miniature thyroid gland after transplantation	Ogundipe etal. (2021)
	Adult	Graves’s disease	EGF, FGF10, Wnt-3a, R-spondin, A83-01, p38 inhibitor, forskolin, TSH	Thyroid organoids for modelling autoimmune disorders	Vaartvan der et al. (2021)
	Fetal	-	EGF, FGF10, retinoic acid, Noggin, R-spondin, A83-01, forskolin, TSH	Derivation of fetal thyroid organoids and modelling of thyroid development	Liang etal. (2022)
Kidney	Pediatric, Adult	BK infection, cystic fibrosis, kidney cancer	EGF, FGF10, R-spondin, A83-01	Derivation of kidney tubule epithelium organoids (tubuloids) from cortical kidney tissue and urine for disease modelling	Schutgens etal. (2019)
	Fetal	-	EGF, FGF7, R-spondin, LDN193189, TTNPB, GDNF, JAK inhibitor I, A83-01, SB202190, CHIR99021	Derivation of ureteric bud organoids from fetal kidney ureteric progenitor cells	Zeng etal. (2021)
Endometrium	Adult	Early pregnancy, endometriosis, endometrial cancer	EGF, FGF10, HGF, Noggin, R-spondin, A83-01	Derivation of endometrial organoids from endometrium and menstrual flow samples for disease modelling	Turco etal. (2017), Cindrova-Davies etal. (2021)
	Adult	-	EGF, FGF10, Wnt-3a, Noggin, R-spondin, A83-01, SB202190, Estradiol	Endometrial organoids from endometrial epithelium respond to hormones	Boretto etal. (2017)
	Adult	Endometriosis, hyperplasia, endometrial cancer	EGF, FGF2, FGF10, Noggin, R-spondin, A83-01, SB202190, Estradiol	Patient-derived organoids for modelling a variety of endometrial disorders	Boretto etal. (2019)
Cervix	Adult	HSV-1 infection, cervical cancer	EGF, FGF7, FGF10, Wnt, Noggin, R-spondin, A83-01, forskolin, SB202190, Estradiol, CHIR	Derivation of healthy cervical organoids from ecto- and endocervical tissue and Pap brush material	Lõhmussaar et al. (2021)
Fallopian Tube	Adult	-	EGF, FGF10, Wnt-3a, Noggin, R-spondin, SB431542	Derivations of organoids from the fallopian tubes	Kessler etal. (2015), Hoffmann etal. (2020)
Ovary	Adult	Ovarian cancer	EGF, Wnt, Noggin, R-spondin, Neuregulin-1, A83-01, forskolin, hydrocortisone, Estradiol	Ovarian cancer organoids model different subtypes of ovarian cancer	Kopper etal. (2019)
Salivary gland	Adult	-	EGF, FGF2, insulin, dexamethasone	Salivary gland organoids rescue hyposalivation	Pringle etal. (2016)
Lacrimal gland	Adult	-	EGF, FGF10, Noggin, R-spondin, A83-01, forskolin	Lacrimal gland organoids can model tearing	Bannier-Hélaouët et al. (2021)
Mammary gland	Adult		MECGM, FCS, forskolin	Derivation of mammary gland organoids and modelling of branching morphogenesis	Linnemann et al. (2015), Fernández etal. (2021)
Placenta	Fetal	-	EGF, HGF, Noggin, R-spondin, A83-01, CHIR99021, Prostaglandin E2	Derivation and differentiation of trophoblast organoids	Haider etal. (2018)
	Fetal	-	EGF, HGF, FGF2, R-spondin, A83-01, CHIR99021, Prostaglandin E2	Trophoblast organoids resemble placenta villous structures and model the maternal-fetal interaction	Turco etal. (2018)

**TABLE 2:** Summary of relevant bioengineered human tissue-derived organoid models.

Organoids	Age	Bioengineered strategy	Disease model	Key finding	Ref
Lung	Adult	Prime editing	Primary cilia dyskinesia	Repair of patient-specific mutation	Vaart et al. (2021)
	Adult	Free ECM culture and polarity reversion	Primary cilia dyskinesia	Motion analysis of rotating ciliated organoids	Wijesekara et al. (2022)
Stomach	Fetal, Pediatric, Adult	Free ECM culture and polarity reversion	SARS-CoV-2 infection	Easy handling of organoid viral infection	Giobbe et al. (2021)
Intestine	Adult	CRISPR-Cas9 editing	Cystic fibrosis	Repair of *CFTR* gene mutation	Schwank et al. (2013)
	Fetal, Adult	Decellularized intestinal ECM hydrogel	-	Expansion of organoids in a GMP-compliant hydrogel	Giobbe et al. (2019)
	Adult	Microfluidic chip	Cryptosporidium parvum infection	Perfusable intestinal tissue-like structure	Nikolaev et al. (2020)
	Adult	Bioprinting	-	Centimetre-scale functional intestinal tissue	Brassard et al. (2020)
	Adult	Microfluidic chip	-	Formation of villi-like protrusions	Kasendra et al. (2018)
	Adult	Microfluidic chip	-	Co-culture with intestinal microbiota	Jalili-Firoozinezhad et al. (2019)
	Adult	Free ECM culture and polarity reversion	*Salmonella enterica* and *Listeria* monocytogenes infection	Easy handling of organoid viral infection	Co et al. (2019)
	Pediatric	Seeding of organoids in decellularized intestinal scaffold	-	Autologous intestinal graft	Meran et al. (2020)
Liver	Adult	Seeding of cholangiocyte organoids on PGA or collagen scaffold	-	Bile duct replacement	Sampaziotis et al. (2017)
	Adult	Perfusion of cholangiocyte organoids into liver *ex vivo*	-	Injected organoids regenerate bile ducts	Sampaziotis et al. (2021)
	Adult	Multi-organ microfluidic chip	Liver cancer	Study of liver-heart crosstalk and drug response	Zhang et al. (2017)
	Adult	Multi-organ microfluidic chip	-	Study of liver-pancreas crosstalk	Bauer et al. (2017)
Pancreas	Adult	Microfluidic chip	Cystic fibrosis-related diabetes	Real-time biochemical measurements	Shik Mun et al. (2019)
Kidney	Adult	Microfluidic chip	-	Perfusable kidney tubule-like structure	Schutgens et al. (2019)
Mammary gland	Adult	Bioprinting	-	Elongated and branching mammary duct structures	Reid et al. (2018)

**TABLE 3:** Summary of relevant RNA sequencing datasets of human tissue-derived organoid models.

Organ	Age	Organoid Types	Sequencing Technique	Platform	Accession Number	Ref
Lung	Fetal	Fetal lung tip and stalks organoids	Bulk-RNAseq	Illumina HiSeq 1,500	GSE95860	Nikolić et al. (2017)
	Fetal	Bud tip organoids	Sc-RNAseq	Illumina HiSeq 4,000	E-MTAB-8221	Miller et al. (2020)
	Adult	Alveolar type II organoids SARS-CoV2 infected	Bulk-RNAseq	Illumina HiSeq 2,500	EGAD00001006241	Youk et al. (2020)
			Sc-RNAseq	Illumina HiSeq 2,500	EGAD00001006242	
	Adult	Alveolar type II organoids SARS-CoV-2 infected and non-infected	Bulk-RNAseq	HiSeq X Ten	GSE152586	Katsura et al. (2020)
	Adult	Lung organoids Cryptosporidium infected	Bulk-RNAseq	Illumina NextSeq 500	GSE112991	Heo et al. (2018)
	Adult	Airway organoids in differentiation towards ciliated cells	Bulk-RNAseq	Illumina NextSeq 500	GSE158775	Vaart et al. (2021)
	Adult	Distal lung organoids	Sc-RNAseq	Illumina NextSeq 500	GSE106850	Salahudeen et al. (2020)
Oesophagus	Adult	Oesophageal adenocarcinoma organoids	Bulk-RNAseq	Illumina HiSeq 2000	EGAD00001004007	Li et al. (2018)
Stomach	Fetal	Gastric organoids	Bulk-RNAseq	Illumina NovaSeq 6,000	GSE153698	Giobbe et al. (2021)
		Gastric organoids SARS-CoV-2 infected and non-infected	Bulk-RNAseq	Illumina NovaSeq 6,000	GSE153684	
	Pediatric	Gastric organoids	Bulk-RNAseq	Illumina NovaSeq 6,000	GSE153698	
		Gastric organoids SARS-CoV-2 infected and non-infected	Bulk-RNAseq	Illumina NovaSeq 6,000	GSE153684	
	Adult	Gastric organoids	Bulk-RNAseq	Illumina NovaSeq 6,000	GSE184390	
Small and large intestine	Fetal	Small intestine and duodenum epithelial organoids	Bulk-RNAseq	Illumina HiSeq 2,500	GSE101531	Senger et al. (2018)
	Fetal	Duodenum organoids	Bulk-RNAseq	Illumina HiSeq 2,500	E-MTAB-11619	Tsai et al. (2022)
	Fetal	Ileum organoids	Sc-RNAseq	Illumina HiSeq 4,000	E-MTAB-8901	Elmentaite et al. (2020)
	Fetal	Small and large intestine organoids	Bulk-RNAseq	Illumina HiSeq 2,500	E-MTAB-5015	Kraiczy et al. (2019)
	Pediatric	Sigmoid colon and terminal ileum organoids	Bulk-RNAseq	Illumina HiSeq 2,500	E-MTAB-5015	
	Pediatric	Small intestinal organoids	Bulk-RNAseq	Illumina NovaSeq 6,000	GSE135108	Giobbe et al. (2019)
	Pediatric	Terminal ileum organoids from children with and without Crohn’s disease	Sc-RNAseq	Illumina HiSeq 4,000	E-MTAB-8901	Elmentaite et al. (2020)
	Adult	Colonic Adenocarcinoma derived organoids	Bulk-RNAseq	Illumina NextSeq 500	GSE65253	van de Wetering et al. (2015)
	Adult	Small intestinal organoids Cryptosporidium parvum infected	Bulk-RNAseq	Illumina NextSeq 500	GSE112991	Heo et al. (2018)
	Adult	Intestinal organoids SARS-CoV-2 infected and non-infected	Bulk-RNAseq	Illumina NextSeq 500	GSE149312	Lamers et al. (2020)
	Adult	Colorectal cancer organoids	Bulk-RNAseq	Illumina NextSeq 500	GSE148347	Brandenberg et al. (2020)
	Adult	Intestinal organoids	Bulk-RNAseq	HiSeq X Ten	GSE163706	Sugimoto et al. (2021)
	Adult	Ileum organoids	Sc-RNAseq	Illumina HiSeq 4,000	GSE119969	Fujii et al. (2018)
Liver	Fetal	Hepatocytes organoids	Bulk-RNAseq	Illumina NextSeq 500	GSE138611	Giobbe et al. (2021)
	Fetal	Hepatocytes organoids	Bulk-RNAseq	Illumina NextSeq 500	GSE111301	Hu et al. (2018)
			Sc-RNAseq	Illumina NextSeq 500	GSE111301	
	Adult	Hepatocytes and cholangiocytes organoids	Bulk-RNAseq	Illumina NextSeq 500	GSE111301	
	Adult	Hepatocytes and liver ductal organoids	Bulk-RNAseq	Illumina NextSeq 500	GSE138611	Giobbe et al. (2019)
	Adult	Liver and liver cancer organoids	Bulk-RNAseq	Illumina HiSeq 1,500	GSE84073	Broutier et al. (2017)
	Adult	Intrahepatic and extrahepatic cholangiocytes organoids	Sc-RNAseq	HiSeq4000	E-MTAB-8495	Sampaziotis et al. (2021)
Pancreas	Fetal	Pancreatic organoids	Bulk-RNAseq	Illumina NextSeq 500	GSE108854	Loomans et al. (2018)
	Adult	Pancreatic organoids	Bulk-RNAseq	Illumina NextSeq 500	GSE108854	
Prostate	Adult	Neuroendocrine prostate cancer organoids	Bulk-RNAseq	Illumina HiSeq 2,500	GSE112830	Puca et al. (2018)
Bladder	Adult	Bladder tumour organoids	Bulk-RNAseq	Illumina HiSeq 2,500	GSE103990	Lee et al. (2018)
Thyroid	Adult	Thyroid follicular cells organoids	Bulk-RNAseq	Illumina HiSeq 2,500	GSE183961	Vaartvan der et al. (2021)
Kidney	Adult	Kidney tubuloids	Bulk-RNAseq	Illumina NextSeq 500	GSE107793	Schutgens et al. (2019)
			Sc-RNAseq	Illumina NextSeq 500	GSE107794	
Endometrium	Adult	Hormone treated and non-treated endometrial epithelial organoids	Bulk-RNAseq	Illumina NextSeq 500	GSE136795	Fitzgerald et al. (2019)
		Hormone treated endometrial epithelial organoids	Sc-RNAseq	Illumina NextSeq 500	Dataset_S02 (supplementary information)	
	Adult	Healthy, entopic, and ectopic endometrial organoids	Bulk-RNAseq	Illumina NextSeq 500	GSE118928	Boretto et al. (2019)
	Adult	Endometrial gland and menstrual blood organoids	Bulk-RNAseq	Illumina NextSeq 500	E-MTAB-9284	Cindrova-Davies et al. (2021)
Ovary	Adult	Ovarian cancer organoids	Bulk-RNAseq	Illumina NextSeq	EGAS00001003073	Kopper et al. (2019)
Lacrimal gland	Adult	Lacrimal gland organoids	Sc-RNAseq	Illumina NextSeq 500	GSE164403	Bannier-Hélaouët et al. (2021)
Placenta	Fetal	Cytotrophoblast organoids	Bulk-RNAseq	Illumina HiSeq 2,500	GSE109976	Haider et al. (2018)

## Lung, upper airways and tonsils

Different stem cell pools reside in the lung epithelium (Rock et al., 2009; Barkauskas et al., 2013), which is canonically divided into airway and alveolar. An early study identified a subpopulation of P63^+^ basal cells as human lung-resident multipotent stem cells capable of forming self-renewing bronchospheres, also containing differentiated cells (Rock et al., 2009). The presence of airway basal cells in the human bronchi was also confirmed by another bronchosphere *in vitro* model (Danahay et al., 2015). In 3D culture, these airway progenitor cells differentiate analogously to the human airway epithelium, giving rise to MUC5AC^+^ goblet cells and acetylated α-tubulin^+^ ciliated cells. However, these models could be kept in culture only for a short time, limiting their use for studying lung development and disease progression *ex vivo.* In a pioneering study, Nikolić and colleagues were able to expand self-renewing primary lung organoids starting from human embryonic and fetal lung epithelial tips (Nikolić et al., 2017). Distal tip organoids consist of a lumen and a single epithelial layer, branched within 10 days, and retained *SOX2* and *SOX9* expression over multiple passages. Moreover, they expressed lung- and tip-specific transcription factors such as *NKX2-1* and *ETV5*. Using an established human airway differentiation medium, tip organoids also partially differentiated into bronchioalveolar structures. This system provided a proof of concept for studying human lung development in a dish. More recently, cultures of human bud tip organoids have been used to study the molecular mechanisms controlling human lung development, identifying SMAD signalling, a key regulator of differentiation into proximal airway epithelium in the developing lung. Moreover, single-cell RNA sequencing (scRNA-Seq) revealed high similarity between primary organoids and *in vivo* airways (Miller et al., 2020). Consistently, human pseudostratified airway organoids were established from adult bronchoalveolar resections and lavage fluid. These consisted of lung differentiated cells, such as basal, ciliated, mucus-producing, and club cells (Sachs et al., 2019). In this study the authors used this system to model a variety of lung diseases, such as cystic fibrosis (CF) and human lung cancer resections. This *in vitro* model was also tested to support respiratory syncytial virus (RSV) infection, demonstrating the versatility of the system. Lung organoids from human primary lung samples have also been used to gain knowledge on the viral pathogenesis of other infectious agents such as SARS-CoV-2 (Katsura et al., 2020; Youk et al., 2020) and influenza virus (Zhou et al., 2018). Within the distal lung epithelium, a subset of alveolar epithelial progenitors was isolated directly from primary human lung and cultured as 3D organoids (Zacharias et al., 2018). Although the formation of alveolar organoids was achieved, a co-culture system with a stromal cell line was required for their establishment, and long-term expansion was not proven. The authors reported that inhibition of the Wnt pathway promoted differentiation towards alveolar epithelial type I (AT1) cell, whereas its activation promoted AT2 cell formation. A later study confirmed the possibility of deriving organoids from human distal lung progenitors (either AT2 or KRT5^+^ basal cells). These were utilised for modelling SARS-CoV-2 infection, as well as for validating the presence and function of a novel progenitor cell subtype (Salahudeen et al., 2020). Importantly, two recent works have also demonstrated the potential of primary lung organoids for investigating the regenerative capability of novel progenitor cells that reside in the lung (Basil et al., 2022; Kadur et al., 2022). Along the upper respiratory tract, organoids can be derived from healthy and diseased human oral mucosa (Driehuis et al., 2019). These organoids can be efficiently infected with herpes simplex virus (HSV) and human papillomavirus (HPV) recapitulating disease mechanisms. Lastly, nasal airway cells isolated from primary ciliary dyskinesia (PCD) patients were three-dimensionally cultured to establish airway organoid lines (Vaart et al., 2021). The latter can be used for modelling this genetic condition *in vitro,* as well as for repairing patient-specific mutations using gene editing, demonstrating the value of primary organoids as an autologous cell source for regenerative medicine. In the context of bioengineering strategies, primary airway organoids were recently engineered for investigating respiratory diseases such as ciliopathies. In particular, an ECM-free culture approach allowed the generation of floating apical-out airway organoids from healthy and PCD cells. Rotational motion of organoids could be evaluated, demonstrating a functional readout that can be used for assessing patient-specific cilia defects (Wijesekara et al., 2022). The development of organoids from human tonsil tissue has been reported very recently (Kim et al., 2022). In this study, purified tonsil epithelial cells were efficiently cultured in 3D and expanded as organoids in an optimised tonsil organoid medium. Tonsil epithelial cell-derived organoids appear phenotypically similar to the human tonsil epithelium. They are indeed composed of stratified squamous epithelial layers and display typical tonsillar biomarkers such as NGFR, CD44 (basal layer cells), MUC1 and CK7 (suprabasal layer cells). Furthermore, to examine the applicability as a functional *ex-vivo* disease model, tonsil organoids were infected with SARS-CoV-2. Tonsil organoids were found to be responsive to the viral infection and could be used to evaluate the therapeutic efficacy of antiviral treatment, thus demonstrating their relevance as a disease modelling and drug testing platform (Kim et al., 2022).

## Oesophagus

Epithelial organoids were first generated from human biopsy specimens of Barrett’s oesophagus, a condition which affects the oesophageal tissue and causes dysplasia and adenocarcinoma. Oesophageal organoids generated from patients with Barrett’s oesophagus displayed cellular hallmarks of the condition, such as the presence of intestinal goblet cells. The addition of FGF10 enabled these organoids to be maintained for months, whereas healthy oesophageal organoids could not be passaged (Sato et al., 2011). Another study also investigating this condition using primary oesophageal organoids highlighted that a transitional epithelium containing basal progenitors is present within the human gastro-oesophageal junction and its expansion is associated with disease progression (Jiang et al., 2017). Interestingly, Nakagawa and colleagues developed a different culture method, demonstrating that biopsy-derived 3D oesophageal organoids can be used to model inflammatory conditions such as eosinophilic oesophagitis (Kasagi et al., 2018). Indeed, co-culture with non-epithelial cells (e.g., immune, or mesenchymal cells) could improve the usefulness of this model and help to gain insights into the pathogenesis of this disease.

## Stomach

Activation of the *LGR5* ^*+*^ stem cells drive self-renewal and regeneration of human gastric glands. Therefore, *in vitro* isolated gastric glands/stem cells can give rise to organoids that mimic the anatomy and physiology of the stomach (Barker et al., 2010). Adult gastric organoids have been generated and expanded from healthy patients to model a common stomach infection *ex vivo* (Bartfeld et al., 2015). These gastric organoids retained morphological characteristics of the human gastric mucosa, and contained differentiated cell subtypes such as chief, enteroendocrine and mucus cells. Furthermore, by microinjecting bacteria into the organoid’s lumen, a host/pathogen co-culture was established, resulting in a high-fidelity model of acute *Helicobacter pylori* infection, and subsequent epithelial inflammatory response. Interestingly, primary gastric organoids with reversed apical-basal polarity were also tested as a model to study enteropathogens such as *Salmonella enterica* and *Lysteria monocytogenes* (Co et al., 2019). More recently, gastric organoids were established from infants and paediatric biopsies to study gastric epithelial homeostasis and disease in children (Jones et al., 2021). Paediatric gastric organoids retain expression of mature markers and can be exponentially expanded, providing a new tool for cell-based postnatal therapies. Similarly, derivation, long-term culture, and characterization of human fetal gastric organoids across different developmental stages has been achieved. Fetal, paediatric, and adult-stage gastric organoids were infected with SARS-CoV-2. Gastric organoids support viral replication, and transcriptomics data highlight comparable profiles with clinical features of COVID-19 patients, providing additional insights into the gastrointestinal transmission of COVID-19 (Giobbe et al., 2021). One of the greatest challenges for stomach researchers is to obtain in culture a population of stable and fully differentiated parietal cells, which produce and secrete acid, as occurs physiologically in the stomach. However, to date, their presence has not been reported in any of the primary gastric organoid models and will probably require more sophisticated 3D systems considering multiple cellular types and including the mesenchymal component.

## Intestine

Intestinal organoids were the first to be developed and have generated an extensive literature output, reporting their use both *in vitro* and *in vivo*. The first report on the development of adult stem cell-derived organoids described the generation of mouse intestinal 3D units with a functional crypt-villus axis from single isolated *Lgr5* ^*+*^ stem cells without the need for a supporting mesenchyme (Sato et al., 2009). Intestinal stem cells expand as organoids with a lumen, crypt-like structures and differentiated cells. These organoids have been successfully derived from healthy and diseased human primary gastrointestinal tissues (Jung et al., 2011; Sato et al., 2011), proving their versatility as a tool to study gut development, regeneration, disease, and tissue engineering. Moreover, to extend the understanding of age-related diseases, primary organoids from adult small intestine and colon were used to perform genome wide mutational signature analysis (Blokzijl et al., 2016). This system enabled the generation of a database of somatic mutations acquired by adult stem cells through aging. Importantly, fetal human intestinal epithelium can also form organoids that can be propagated long-term (Fordham et al., 2013). Recently, single cell profiling of human fetal intestinal organoids provided the opportunity to investigate the dynamic changes and epigenetic mechanisms of gut epithelial cells occurring during fetal development (Kraiczy et al., 2019; Elmentaite et al., 2020). These platforms can be used to model genetic diseases and investigate treatments. Indeed, the functionality of the CFTR protein (mutated in cystic fibrosis patients) has been assessed in primary cystic fibrosis intestinal organoids (Dekkers et al., 2013). Furthermore, a pivotal study described the CRISPR-Cas9-mediated correction of the F508 deletion in the CFTR gene in adult intestinal stem cells derived from cystic fibrosis patients (Schwank et al., 2013). This demonstrated the enormous potential of genome editing when applied to primary organoids. To further extend the potential of organoids in disease modelling and repair, human intestinal organoids were recently used to compare efficiency and specificity of different genome editing technologies such as prime editing and base editors (Geurts et al., 2021). Human intestinal organoids proved to be useful in modelling *ex vivo* inflammatory bowel diseases (IBD) such as ulcerative colitis and Crohn’s disease (Arnauts et al., 2020), which pathogenetic mechanisms are difficult to reproduce in animal models. Finally, intestinal organoids can also be infected with various viral and bacterial pathogens and used for microbiological and pharmacological studies (Heo et al., 2018; Co et al., 2019; Lamers et al., 2020; Zhou et al., 2020). However, their 3D structure is quite elemental, and progress has been made in the culturing of organoids, including intestinal ones, into more complex structures. The need for developing different 3D ECMs that support organoid expansion is not only related to accurate biomimicry of normal physiology, but also for improving their clinical application (Wang et al., 2018). A recent demonstration indicates that ECM gels derived from decellularized small intestinal mucosa can be employed as a 3D scaffold for growing a variety of primary human organoids, comprising fetal and adult intestinal organoids (Giobbe et al., 2019). Using primary organoids combined with different technologies, other studies have attempted to model intestinal morphogenesis and stem cell self-organization *in vitro*. As an example, Lultolf’s lab focused on using micro-engineered cultures (Brandenberg et al., 2020; Nikolaev et al., 2020) and bioprinting technology (Brassard et al., 2020). These intestinal organoids-on-a-chip develop into functional and perfusable tube-like structures which efficiently mimic the geometry of the native crypts and preserve the homeostatic and regenerative capacity of the human gut (Nikolaev et al., 2020). The same group has also demonstrated that is possible to sequentially bioprint large-scale tissues, including primary human intestinal organoids, to ultimately recreate segments of the gastrointestinal tract *in vitro* (Brassard et al., 2020). Key research from Ingber’s lab have also shown that cells dissociated from human small intestinal organoids are able of forming complex epithelial structures when seeded into a microfluidic device (Kasendra et al., 2018; Jalili-Firoozinezhad et al., 2019). The gut chips replicate several characteristics of the native small intestine such as elongated villi containing differentiated cells, brush border digestive enzyme activity, and an apical lumen (Kasendra et al., 2018). Furthermore, they demonstrate that the intestine-on-a-chip makes it feasible to co-culture intestinal epithelium with aerobic and anaerobic human gut bacteria, enabling the possibility of studying the human intestinal microbiome *in vitro* (Jalili-Firoozinezhad et al., 2019). From a regenerative medicine perspective, it has been shown that human adult stem cell-derived large intestinal organoids can be used to successfully reconstruct the colon epithelium *in vivo* (Sugimoto et al., 2018). In this direction, primary organoids can be reliably derived from children with short bowel syndrome (SBS) and intestinal failure (IF) and used to generate a functional and transplantable engineered small intestinal mucosa (Clevers et al., 2019; Meran et al., 2020). These findings demonstrate that bioengineering strategies are key for improving organoid complexity and functional maturation. While further research is required to better improve organoids maturation (Sugimoto et al., 2021) and promote their expansion in GMP-compatible conditions to enable their clinical application, intestinal organoids are among the closest to an organoid-based regenerative strategy with a very recent disclosure of their first transplantation in human.

## Liver and biliary tree

Since the discovery that *Lgr5* ^*+*^ stem cells are also present near the bile ducts, and can form epithelial organoids (Huch et al., 2013a), protocols for deriving and culturing human primary liver organoids have been established. Pioneering work led by the Clevers’ group reported that human primary ductal cells (from intrahepatic bile ducts) can be reliably cultured long-term as 3D epithelial organoids (Huch et al., 2015). These adult stem cell-derived liver organoids express both biliary and hepatic markers. By changing the medium conditions these can differentiate to functional hepatocytes that produce albumin and secrete bile acids. Additionally, these organoids maintain a functional hepatic phenotype when transplanted into the damaged liver of immune-deficient mice. The authors further demonstrated that organoids derived from patients affected by α1-antitrypsin (A1AT) deficiency and Alagille syndrome (AGS) can model the respective inherited hepatobiliary disorders. In detail, they observed reduced secretion as well as aggregates of misfolded A1AT in the hepatocyte within the organoids. On the other hand, organoids from AGS samples present the structural malformations found in the biliary tree of these patients and are unable to fully express biliary markers upon differentiation (Huch et al., 2015). Epithelial organoids can be also established from single healthy fetal, pediatric and adult hepatocytes, thus demonstrating the proliferative and regenerative properties not only of biliary tree cells, but also of hepatic ones (Hu et al., 2018). Liver organoids (comprising ductal and hepatocyte organoid lines) can proliferate long-term and display some key functional aspects such as albumin secretion, A1AT secretion, CYP3A activity, and integration in a regenerating liver (Hu et al., 2018). Alternatively, several studies have shown that liver organoids can be derived from extrahepatic primary tissues. Notably, culture conditions using R-spondin and Noggin similar to that described above, support the growth of human gallbladder organoids, that can be propagated for months and express typical biliary cells markers (Lugli et al., 2016). Adopting different culture conditions, another pivotal study demonstrated that primary human extrahepatic duct cells can form extrahepatic cholangiocyte organoids (Sampaziotis et al., 2017). The resulting organoids possess the same morphology and function as primary cholangiocytes and when seeded on a polyglycolic acid (PGA) or collagen scaffold, they proliferate and expand around the synthetic polymer. Moreover, upon transplantation into a mouse model of bile duct disorder, the bioengineered construct efficiently exhibits biological features and function of biliary tissue. More recently, Sampaziotis and colleagues employed human primary extrahepatic cholangiocyte organoids to regenerate the intrahepatic bile ducts of the human liver using an *ex vivo* perfusion system (Sampaziotis et al., 2021). Cholangiocytes organoids were delivered through the bile ducts of human donor livers, and efficiently engrafted in the intrahepatic biliary tree. Transplanted organoids regenerated up to 85% of the injected ducts, upregulated key biliary and intrahepatic markers, and preserved function (Sampaziotis et al., 2021). This innovative investigation has provided strong preliminary evidence that primary cholangiocyte organoids might be used to bioengineer human biliary epithelium in a translational setting. Bioengineering solutions have proven successful in the context of liver-on-a-chip. For instance, a multimodal microfluidic chip with integrated liver spheroids seems to be a reliable and automated way to control and monitor in culture several parameters such as flow, pH, O2, temperature, and electrochemical sensors for the evaluation of protein biomarkers (Zhang et al., 2017). In a further study, the functional combination of liver spheroids and pancreatic islets in a microfluidic platform was reported. Co-culture of the two components allowed the investigation of the physiological cross-talk between liver and pancreas and the assessment of glucose circulation between the two tissue constructs (Bauer et al., 2017).

## Pancreas

Primary organoids have been widely used to model human pancreatic development and disease. After the first establishment of long-term, self-renewing adult mouse pancreatic duct organoids (Huch et al., 2013b), the protocol was adapted to generate organoids from human fetal (Bonfanti et al., 2015) and adult pancreatic progenitors (Boj et al., 2015). Human fetal pancreatic organoids resemble 3D duct-like structures which maintain fetal features such as the expression of pancreatic progenitor markers (i.e., *PDX1*, *NKX6-1*, *SOX9*). Importantly, modulation of EGF signalling impacted organoid proliferation and differentiation into endocrine cells (Bonfanti et al., 2015). Adult human pancreatic tissue was also used to derive and expand 3D pancreatic ductal organoids and explore pancreas development and self-renewal (Loomans et al., 2018). These organoids are genetically stable in culture and contain progenitor cells localised within the tips of the budding compartments, which are capable of differentiating, although incompletely, to the endocrine lineage. Intriguingly, after transplantation under the kidney capsule of immunodeficient mice, a few functional insulin-producing cells could be observed. Pancreatic ductal organoids can be a source of patient-derived pancreatic ductal epithelial cells that, when bioengineered in a microfluidic chip, are capable of modelling cystic fibrosis-related diabetes. Importantly, multiple analyses such as functional assays (i.e., CFTR channel activity) and biochemical measurements (i.e., glucose and insulin concentrations) could be performed in real-time (Shik Mun et al., 2019). Lastly, bioengineering of pancreatic islets has always shown potential, especially as a strategy for β cell replacement therapy for diabetes. Therefore, further efforts are needed to translate these complex *in vitro* systems into clinical practice, due to their lack of mature and functional endocrine cells (Bakhti et al., 2018; Siehler et al., 2021).

## Prostate and bladder

Cultures of prostate organoid have been established either from normal or diseased human prostate samples. In 2014, Karthaus and colleagues published a protocol for long-term expansion of human prostate organoids (Karthaus et al., 2014). Such organoids were derived from both single primary luminal or basal cells, were genetically stable in culture over time, and were androgen responsive, thus closely recapitulating morphology and physiology of glandular tissue. Notably, organoids derived from luminal cells strongly expressed luminal markers such as *NKX3-1* and PSA compared to basal-derived organoids, consistent with their *in vivo* histological counterpart. A similar method of isolation, with slightly different culture conditions was applied to create human bladder organoids. Urinary bladder epithelium (i.e., urothelium) can be efficiently propagated *in vitro* as 3D organoid and used to study urothelial cancer when derived from patient-specific primary tissue (Mullenders et al., 2019).

## Thyroid

Some recent studies have shown the generation of thyroid organoids from human primary thyroid gland tissue (Ogundipe et al., 2021; Vaartvan der et al., 2021). These self-renewing organoids were used to investigate the stemness of thyroid gland cells and could be maintained in culture for long-term. Thyroid follicular organoids possessed the phenotypic and genetic characteristics of follicular epithelium and contained differentiated follicular cells. Furthermore, thyroid organoid cells transplanted into a mouse model of hypothyroidism led to the formation of functional hormone-producing thyroid-like follicles (Ogundipe et al., 2021). A following study reported similar findings and described how primary thyroid organoids can model autoimmune disorders such as Graves’ disease (Vaartvan der et al., 2021). Very recently, long-term culture of human fetal thyroid organoids has been established. By combining transcriptomic data and fetal thyroid organoid systems, this study highlighted the potential of organoid technology for exploring human thyroid gland development *ex vivo* (Liang et al., 2022).

## Kidney

Only recently, have a few reports shown the development of human kidney organoids from primary renal tissue. In 2019, Clevers and colleagues first demonstrated that human cortical kidney tissue can be used to reliably establish renal organoids, termed tubuloids, which physiologically model homeostasis and disease of the tubular epithelium (Schutgens et al., 2019). Although tubuloids lack glomerular cells, scRNA-Seq revealed that they are composed of different nephron compartments such as proximal tubules, loop of Henle, distal tubules and collecting duct. Proximal tubule transporter function was also evaluated by assessing the activity of the xenobiotics efflux pump ABCB1, which upon inhibition triggers tubular cells to retain fluorescent calcein intracellularly. To gain insights into kidney disease, tubuloids were used to model *ex vivo* a common viral infection of the kidney (BK virus). In addition, they were successfully established from Wilms tumour paediatric specimens. Moreover, it has been shown that it is possible to generate tubuloids from human urine, an extremely accessible and non-invasive approach to produce organoids. The latter can also be derived from the urine of cystic fibrosis patients, enabling the development of an *in vitro* platform for personalised disease modelling and drug screening (Schutgens et al., 2019). In addition to these findings, tubuloids were cultured on ECM-coated microfluidic chip to further promote tubule formation and grow engineered perfusable kidney tubules (Schutgens et al., 2019). These recent data suggest that increasingly complex and bioengineered culture systems are crucial to support and aid the development of a functional and mature nephron unit model. More recently, human fetal kidneys were utilised to isolate ureteric bud progenitor cells to finally establish optimal culture conditions for generating a renal organoid model derived from primary fetal samples (Zeng et al., 2021). Overall, this 3D *in vitro* system allowed a better understanding of kidney branching morphogenesis, as well as congenital defects.

## Endometrium, cervix, and fallopian tube

Adult endometrial specimens have been utilised to derive primary human endometrial organoids, by adapting the culture conditions described for the generation of intestinal and liver organoids (Boretto et al., 2017; Turco et al., 2017). Human endometrium-derived organoids can be cultured long-term and generate glandular structures which morphologically and molecularly resemble the human endometrium. Moreover, they are functionally responsive to ovarian hormones (oestrogen and progesterone). When stimulated with prolactin and placental hormones (chorionic gonadotropin and placental lactogen) they differentiate to a decidual-like phenotype characteristic of early pregnancy (Turco et al., 2017). Interestingly, scRNA-Seq confirmed the presence of a higher number of differentiated cells in the endometrial organoids after hormonal stimulation (Fitzgerald et al., 2019). To explore pathological conditions of the human uterus, such as endometriosis and endometrial cancer, organoids have been reproducibly derived from multiple primary biopsy samples (Boretto et al., 2019). These patient-specific organoids maintain the cellular pathophysiological characteristics of the diseased tissue and can be employed to study the mutational landscape of endometrial cancer and test chemotherapeutic compounds. More recently, endometrial organoids were derived from gland fragments retrieved from menstrual flow samples (Cindrova-Davies et al., 2021). Similar to the urine-derived tubuloids (Schutgens et al., 2019), this method shows a non-invasive way to build an innovative 3D *in vitro* model of the human endometrium. Overall, endometrial organoids provide an *ex vivo* system to reliably study menstrual cycle, pregnancy, and endometrial diseases. In addition to endometrial organoids, a recent study showed that patient-derived organoids can also be derived from the cervix to model homeostasis and disease of the cervical tissue (Lõhmussaar et al., 2021). In particular, it is possible to successfully generate 3D cervical epithelial organoids from PAP brush samples. These organoids can be infected with herpes simplex virus type 1 (HSV-1) to model this sexually transmitted infection. Additionally, using the same starting material derived from cervical cancer patients, cervical tumoroids were generated. The latter recapitulates the tumour-specific gene expression and phenotype, also showing presence of viral integration of human papilloma virus (HPV), possibly reflecting human cervical carcinogenesis (Lõhmussaar et al., 2021). Primary organoids were also derived from the fallopian tubes (Kessler et al., 2015; Kopper et al., 2019; Hoffmann et al., 2020). Following TGF-β signalling inhibition and Wnt pathway stimulation, adult fallopian epithelial cells expand three-dimensionally and form cystic structures that can be cultured long-term. Both ciliated and secretory cells were detected within the fallopian tube organoids, showing similar morphological and functional features of the native epithelium (Kessler et al., 2015). Physiological and pathological mechanisms arising during fetal development as well as early pregnancy mechanisms could be studied using these systems to recapitulate the physiological uterine microenvironment in a dish.

## Ovary and testis

Epithelial organoids from human ovaries were efficiently cultured long-term for the first time in 2019 (Kopper et al., 2019). Although this study focussed on the generation and characterisation of ovarian cancer organoids, healthy ones were also successfully derived from the ovarian surface epithelium (Mullenders et al., 2019). With regards to the male gonads, although some studies have reported self-organisation of testicular cells into 3D aggregates (Baert et al., 2017; Sakib et al., 2019), successful long-term expansion, and functional maturation of human testicular organoids in chemically defined media and 3D matrices has not yet been reported. In this light, more physiological culture systems and bioengineered approaches that may help recapitulate human gonads development are needed to improve these 3D models, with the goal of generating haploid cells *in vitro*.

## Salivary and lacrimal glands

Protocols for the expansion of human adult stem/progenitor cells into organoids have been reported for both glands. Coppes and colleagues described the 3D culture of human salivary gland (SG) organoids from primary submandibular SG biopsies (Pringle et al., 2016). Although these organoids contained differentiated salivary gland cell types, the culture conditions depended solely on EGF and FGF and were not optimal for long-term expansion and self-renewal. However, upon transplantation into irradiated murine submandibular glands, these organoids efficiently engrafted and rescued the salivary gland function *in vivo* (Pringle et al., 2016). This study highlighted the potential of organoid-based cell therapy for the treatment of severe hyposalivation conditions. Human salivary gland stem cells were also cultured in bioengineered microwells to foster 3D spheroids differentiation and function (Shin et al., 2018). The resulting organoids retained salivary epithelial cell components such as acinar and ductal cells that displayed upregulated mRNA expression levels of salivary epithelial markers. Although few studies over the last years attempted to bioengineer the lacrimal gland, only a single recent publication has described the derivation and long-term culture of human lacrimal gland organoids (Bannier-Hélaouët et al., 2021). Dissociated tissue fragments from diagnostic lacrimal gland biopsies can be expanded as 3D organoids with 100% efficiency while maintaining the epithelial identity of the primary tissue. Furthermore, when exposed to differentiation medium, organoids showed increased expression of epithelial and functional lacrimation markers, as further confirmed *via* scRNA-Seq.

## Mammary gland

Breast epithelium was one of the first tissues used to advance research into 3D culture systems back in the 1987 (Li et al., 1987). However, organoid models resembling the ductal and lobular units of the mammary gland have only recently been developed. Linnemann *et al.* generated 3D branching epithelial structures from freshly isolated human mammary epithelial cells cultured in floating collagen gels (Linnemann et al., 2015). Even though these organoids could not be cultured for more than three passages, they contained specialised differentiated cells and exhibited contractility - a functional feature of the mammary ducts during lactation. Interestingly, a recent work highlighted the potential of primary mammary organoids to investigate complex developmental processes such as branching morphogenesis (Fernández et al., 2021). In a similar manner to that reported for the intestine, human mammary gland epithelial cells were sequentially bioprinted to allow the generation of spatially defined self-assembled mammary organoids. These could consistently form a contiguous duct-like lumen and be also bioprinted in a collagen hydrogel. This work suggests that bioengineered strategies may take over from standard 2D and 3D cultures in order to streamline efficiency, reproducibility, and scalability (Reid et al., 2018).

## Placenta

The placenta is the only extraembryonic tissue from which primary organoids have been generated so far. 3D culture of human trophoblast cells enables the development of trophoblast organoids that closely resemble the complex branched morphology of the human placental epithelium (Haider et al., 2018; Turco et al., 2018). Human placenta explants can be used to isolate trophoblast stem cells which are further cultured in a 3D ECM environment that allows their three-dimensional growth. Long-term culture of human trophoblast organoids can be achieved in chemically defined culture conditions eliciting EGF and FGF signalling’s activation and TGF-β inhibition. These organoids resemble the human first trimester placenta, showing similar transcriptomic and methylation profiles (Turco et al., 2018). Furthermore, organoids differentiated towards syncytiotrophoblast and extravillous trophoblast lineages can be obtained. Trophoblast organoids are a powerful model tool to study human placenta development and disease *in vitro*. However, further work is essential to develop more complex systems that can fully recapitulate the cell heterogeneity and function of placenta for the establishment and maintenance of pregnancy, to allow an accurate investigation of the fetal-maternal interface.

## Discussion

The main appeal of primary cellular models is to recapitulate the human tissue more accurately, but their use has been historically limited by the difficulties of recapitulating its complexities *in vitro*. The limited representation of rarely occurring cell types, and the failure to account for cell-to-cell interaction and represent functional differentiation has limited the potential of these approaches. Since 2009, following Sato’s publication (Sato et al., 2009), organoids were introduced to the research community, and rapidly became a unique modelling tool. To date, several studies have highlighted the importance of developing multicellular three-dimensional *in vitro* models, either for investigating tissue homeostasis and disease, or for easily screening for new drugs. With the recent advances in basic and translational research, the organoid technology demonstrated its unprecedented potential. Indeed, it has already been discussed elsewhere that organoids have the ability to better resemble tissue-like morphological and functional features over traditional 2D cell cultures (Li et al., 2019; Corrò et al., 2020). In fact, given their cellular complexity, organoids are suitable for modelling human organogenesis and diseases, whereas 2D systems lack of that complexity that would allow tissue-level studies. Another aspect to be considered is that organoids, compared to both 2D cultures and animal models, recapitulate patient-specific pathological cues and therefore it is possible to investigate the biological functions associated with a specific abnormal/altered cell type. While epithelial derived organoids are the most investigated, 3D culture systems have been subsequently described for different cell types. In particular, primary human organoids have been derived from fetal and adult human tissues originating from different developmental compartments: endoderm, mesoderm, ectoderm, and extraembryonic tissues (Figure 2A; Table 1). Having the potential to recapitulate physiological mechanisms of their tissue of origin both in healthy and pathological conditions, primary organoids allow in depth studies of normal and diseased tissues. These systems have been used to investigate, for example, stem/progenitor cell biology, disease mechanisms, and experimental therapies. One of the main applications in which primary organoids show the greatest potential is for disease modelling. The high tissue- and patient-specificity of primary organoids, as well as their suitability for genetic manipulation or *ex vivo* screening, makes this an irreplaceable technique. The combination of single-cell profiling, bioengineering, and organoids modelling provided an unprecedented platform for resolving human organ development and regeneration. Moreover, applying organoid technology to regenerative medicine, developing innovative functional treatments and to expand our understanding of rarely occurring cell types, became a tangible possibility. Clinically relevant organoids are therefore needed for providing transplantable tissues ‘in-a-dish’. For instance, innovative ECMs and chemically defined media for culturing organoids, play a crucial role in providing GMP-compliant products that can avoid immune rejection upon transplantation. Reproducibility of organoid generation, as well as of morphology and function, is a limitation that still needs to be addressed. Robust pipelines for the development, characterization and ultimately clinical translation of organoids must be designed and disseminated. In this direction, the organoid research community is moving towards establishing standards for data quality and reproducibility in the context of organoid development and applications. For instance, variability between organoids derived from different patients and intra-organoid heterogeneity needs to be considered. To improve these standards, statistical reproducibility should be robust in the sense of repeated replicates in at least three independent biological replicates (derivation of organoids from at least three independent patients) (Zhao et al., 2022). Furthermore, it is crucial to conduct a comprehensive characterization of the organoids at the gene and protein levels. Gene expression can be easily evaluated with standard techniques (real-time PCR, RNA Sequencing), while protein content can be investigated using also well-established methodologies such as flow cytometry, immunofluorescence, Western blotting, ELISA. Lastly, to confirm the tissue of origin, appropriate controls (e.g., fetal, pediatric or adult tissue) should be used whenever possible. For the further improvement and scalability of organoid technology, bioengineering approaches seem to be the way forward. Co-culture, microfluidics and bioprinting are candidate strategies to achieve this goal. Engineering the stem cell niche and the ECM microenvironment is fundamental for the growth of reliable and fully functional organoid models. This will substantially advance not only the physiological level of organoid maturation, but also reproducible generation in the framework of disease modelling and clinical translation (Garreta et al., 2020; Hofer and Lutolf, 2021). An additional important aspect to consider when developing more complex primary organoid-based systems that better recapitulate human organs, is the inclusion of a vascular compartment. Several groups have attempted at combining organoids with endothelial cell networks using co-culture systems, microfluidics, or *in vivo* transplantation (Takebe et al., 2013; Mansour et al., 2018; Homan et al., 2019). Moreover, an alternative promising strategy is the generation of blood vessel organoids aimed at vascular disease modelling (Wimmer et al., 2019). So far, this research has been limited to pluripotent stem cells and therefore further work is required to establish comparable 3D blood vessel models from primary endothelial cells. Human tissue level maturation of the organoids for obtaining fully specialized and functional cells is still a challenge in this research field. Alongside investigation of the human body, the goal of international networks, such as the Organoid Cell Atlas (Bock et al., 2020), is to achieve the standardization and validation of organoid systems to ultimately promote their clinical and basic research. In conclusion, human primary organoids are an extremely valuable tool for *in vitro* disease modelling and hold great promise in clinical application within the next few years.
